# Biosynthesis of Gold and Silver Nanoparticles Using Phytochemical Compounds

**DOI:** 10.3390/molecules28073240

**Published:** 2023-04-05

**Authors:** Ade Zuhrotun, Dede Jihan Oktaviani, Aliya Nur Hasanah

**Affiliations:** 1 Department of Pharmaceutical Biology, Faculty of Pharmacy, Universitas Padjadjaran, Jalan Raya Bandung-Sumedang KM 21 Jatinangor, Bandung 45363, Indonesia; 2 Department of Pharmaceutical Analysis and Medicinal Chemistry, Faculty of Pharmacy, Universitas Padjadjaran, Jalan Raya Bandung-Sumedang KM 21 Jatinangor, Bandung 45363, Indonesia

**Keywords:** green synthesis, gold nanoparticles, silver nanoparticles, phytochemical compounds

## Abstract

Gold and silver nanoparticles are nanoparticles that have been widely used in various fields and have shown good benefits. The method of nanoparticle biosynthesis utilizing plant extracts, also known as green synthesis, has become a promising method considering the advantages it has compared to other synthesis methods. This review aims to give an overview of the phytochemical compounds in plants used in the synthesis of gold and silver nanoparticles, the nanoparticle properties produced using plant extracts based on the concentration and structure of phytochemical compounds, and their applications. Phytochemical compounds play an important role as reducing agents and stabilizers in the stages of the synthesis of nanoparticles. Polyphenol compounds, reducing sugars, and proteins are the main phytochemical compounds that are responsible for the synthesis of gold and silver nanoparticles. The concentration of phytochemical compounds affects the physical properties, stability, and activity of nanoparticles. This is important to know to be able to overcome limitations in controlling the physical properties of the nanoparticles produced. Based on structure, the phytochemical compounds that have ortho-substituted hydroxyl result in a smaller size and well-defined shape, which can lead to greater activity and stability. Furthermore, the optimal condition of the biosynthesis process is required to gain a successful reaction that includes setting the metal ion concentration, temperature, reaction time, and pH.

## 1. Introduction

Nanoparticles (NPs) are nanoscale particles of various materials and various shapes, with sizes ranging from 10^−9^ to 10^−7^ m [1,2,3]. Nanoparticles made with noble metals, such as gold (AuNPs) and silver (AgNPs), have sparked considerable interest in recent years as ideal materials due to their unique features, such as biocompatibility, inert nature, stability, strong absorption of electromagnetic waves in the visible region by surface plasmon resonance (SPR), and low toxicity [4]. AuNPs and AgNPs are the most prominent nanoparticles that have been extensively used in various fields, both medical and non-medical [5,6,7,8]. For instance, they are used as antimicrobial substances, catalysts, drug delivery carriers, sensors, environmental remediation agents, cosmetic ingredients, and in other applications [9,10,11,12,13,14].

The synthesis of AuNPs and AgNPs is carried out by chemical, physical, and ‘green synthesis’ methods [15,16,17]. Chemical and physical methods are slowly being replaced by green synthesis methods due to some of their shortcomings, such as the release of harmful and toxic chemical compounds, the use of large amounts of energy, the use of tools, and their complex synthesis conditions [18,19,20,21,22,23].

Green synthesis is a method of synthesizing nanoparticles using natural materials that are safe and environmentally friendly. The materials that are mainly used as reducing and stabilizing agents in green chemistry are extracts from various plants, algae, and microorganisms (bacteria, fungi, and yeast) [24,25,26]. The use of the green chemistry method to synthesize nanoparticles has attracted interest in recent times due to some of its advantages [27], such as low energy consumption and being environmentally friendly, pollution-free, non-toxic, cost-effective, and more sustainable [28,29,30,31]. In addition, nanoparticles produced using green chemistry are relatively more stable and safer than those produced using other methods [24,32].

Plants have been known as cost-effective producers of natural chemical compounds and have great potential in the detoxification of heavy metals and toxic materials [33,34]. Because of the phytochemicals contained in their extracts, which function as reducing and stabilizing agents in the nanoparticle synthesis process, some plant parts, such as leaves, flowers, stems, roots, and fruits, have been extensively utilized in the synthesis of various nanoparticles, including AuNPs and AgNPs [25]. However, green synthesis NPs using plant extracts have the limitation of difficult size, shape, and uniformity of metal nanoparticle control due to the variety of phytochemical content in plant extracts. Therefore, an understanding of phytochemical compounds involved in biosynthesis is required, including an understanding of the structure of phytochemical compounds that act as reducing and stabilizing agents [27,35]. Until now, there has been no review discussing the phytochemical compounds contained in plant extracts that are related to the biosynthesis of AuNPs and AgNPs. It is very important to comprehend the main phytochemical compounds, including their structure, that play a role in the biosynthesis of both types of nanoparticles and their influence on the characteristics and activity of the resulting nanoparticles to overcome the main limitation of green synthesis NPs. This review provides an overview of the phytochemical compounds in plants used in the synthesis of AuNPs and AgNPs, the properties of the nanoparticles produced using plant extracts based on the concentration and structure of the phytochemical compounds, and the applications of AuNPs and AgNPs in various fields.

## 2. Synthesis of Gold and Silver Nanoparticles Using Plant Extracts

The concept of ‘green chemistry’ in nanoparticle synthesis is the development of nanoparticles that involves natural materials such as plants, fungi, bacteria, etc., which are environmentally friendly. This development method is hereinafter referred to as ‘green synthesis’ [36]. Among the biological materials mentioned, plants appear to be the best candidates and are appropriate for large-scale nanoparticle biosynthesis. The use of plant extracts in the synthesis of nanoparticles has several advantages over the use of other biological materials, among which the kinetics for synthesis with an approach using plants is much higher than that of other biosynthetic approaches, and is equivalent to the synthesis of nanoparticles using chemical methods [37]. For example, research conducted by Dare et al. [38] has reported that green synthesis utilizing some plants requires a nanoparticle growing time ranging from 2 to 30 min. An additional period of some hours is also required to finish the reaction [39,40]. Meanwhile, the growth of nanoparticles synthesized using microorganisms required a longer time: it takes > 30 min and even up to a few days [41,42,43,44]. Furthermore, the process is environmentally friendly and economical because it can avoid the use of intermediate base groups. The use of plant extracts has also shown great promise in removing toxicity and pollutants from the waste produced by environmentally friendly methods [45]. In addition, compared to the use of microorganisms, the utilization of plant extracts for nanoparticle synthesis is more favourable because plant extracts are easy to obtain and do not require a complicated cell culture maintenance process [46]. Nanoparticles produced through the utilization of plant extracts are also more stable during long-term storage due to the resulting form of non-aggregated nanoparticles [47]. A summary of the advantages and disadvantages of green synthesis of nanoparticles using plant extracts and microorganisms is presented in Table 1.

**Figure 1:**
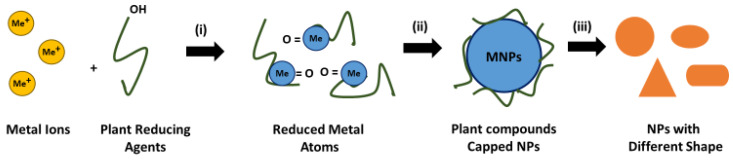
The mechanism of biosynthesis of metal nanoparticles using plant extracts: (i) activation stage, (ii) growth stage, and (iii) termination stage.

Green synthesis utilizing plant extracts has been used for the production of various metal nanoparticles, including those made with silver and gold [48]. Plant parts, such as leaves, roots, bark, etc., are utilized as sources in green synthesis of AuNPs and AgNPs using plants. These plant parts are cut into small pieces and extracted using appropriate solvents, then the extracts are purified by filtration and centrifugation. The resulting extract is mixed with a saline solution of HAuCl_4_ or AgNO_3_, which will cause the reduction of metal ions (Au^3+^/Ag^+^) into atoms (Au^0^/Ag^0^) and form stable nanoparticles in the presence of redox enzymes and metabolites. The solution colour changes indicate AuNP and AgNP formation. Plant extracts act as reductors and capping mediators for the synthesis of AuNPs and AgNPs [47,49,50,51,52]. In general, the mechanism of biosynthesis of metal nanoparticles utilizing plant extracts consists of three main stages, as shown in Figure 1, namely (i) the activation stage, where metal ion reduction and the nucleation of reduced metal atoms occur; (ii) the growth stage, in which small contiguous nanoparticles form larger-sized particles spontaneously accompanied by thermodynamic stabilization; (iii) the termination stage that determines the nanoparticles’ final shape [46].

Based on the initial material used for the preparation process, the methods of synthesizing AuNPs and AgNPs is divided into two, namely the top–bottom method and the bottom–up method [53]. In the top–bottom method, bulk material is broken into nanoparticles using various methods such as milling, attrition, grinding, or etching. This method is more suitable for producing nanoparticles larger than 100 nm. This process involves high energy consumption and can cause imperfections in the surface of nanoparticles so that it will have a significant impact on their physical and chemical properties [22,25,53,54]. Meanwhile, the bottom–up method can produce nanoparticles with fewer faults than the top–bottom method, and produce a homogeneous chemical composition. This method is also cost-effective and can produce a number of important nanoparticle formations in a short time. It is also known as the ‘self-assembly method’, where atomic growth occurs at the nucleation centre to generate nanoparticles. Due to its advantages, the bottom–up method is often used in the biosynthesis of AuNPs and AgNPs [55].

AuNPs and AgNPs generated using plant extracts have been used extensively in various fields, such as in the medical field as antimicrobial and anti-biofilm agents, in anticancer therapy, and as anti-inflammation agents. In addition, Au/AgNPs are also used as catalysts in reduction and oxidation reactions, drug delivery vehicles, and colorimetric detectors. These applications are described in the following sections.

### 2.1. Antimicrobial and Anti-Biofilm Agents

The use of metal nanoparticles, such as AuNPs and AgNPs, can provide a feasible alternative to the present methods of inhibiting the growth of many pathogenic species as novel antimicrobial and anti-biofilm agents. Nanoparticles have antimicrobial activity due to their unique properties, namely a high ratio of surface area to volume, their ultra-fine size, and the presence of biochemical parts on their surface (surface coating or functional groups). These properties determine the therapeutic and adverse effects of the resulting nanoparticles [56]. Table 2 provides information about several nanoparticles that are synthesized utilizing plant extracts that have biological activity as antimicrobial and anti-biofilm agents against pathogens.

It can be seen in Table 2 that green-synthesized AgNPs have been shown to have significant antibacterial activity (bacteriostatic, bactericidal) against Gram-negative bacteria, such as *Pseudomonas aeruginosa* and *Escherichia coli*, as well as Gram-positive bacteria, such as *Staphylococcus aureus*. Meanwhile, green-synthesized AuNPs showed lower antibacterial activity than AgNPs, and some studies, such as [57,58,59], reported no antibacterial activity even at the highest concentrations tested. However, interestingly, green-synthesized AuNPs show as much biofilm inhibitory activity as green-synthesized AgNPs, as evidenced in the research of Singh et al. [59], which showed the presence of anti-biofilm activity at a concentration of 12.5 mg/mL for AuNPs and 6.25 mg/mL for AgNPs. This is possible because AuNPs and AgNPs have a greater affinity for proteins and tend to attach to cell surface proteins [79]. Likewise, studies on the antimycotic activity of green-synthesized NPs have revealed that AgNPs have the best activity against some strains of fungi, while AuNPs show low activity [60,68,69]. The antimicrobial and anti-biofilm properties of these nanoparticles are enhanced by the anchoring of biologically active and biocompatible molecules against the synthesized metal nanoparticles. Biologically active phytochemical compounds that are adsorbed in nanoparticles are bacteriostatic, because they have substitutions of various functional groups, such as -OH, -NH_2_, -COOH, -NO_2_, etc., which play a pivotal role in various types of biological activity. Therefore, the green synthesis of nanoparticles using plant extracts has been proven to show excellent potential for their use as antimicrobial and anti-biofilm agents [60,80]. An example of the application of metal nanoparticles as antimicrobials, specifically for the treatment of infectious conditions, was reported by Luo and colleagues. Through the green synthesis process (G-AgNPs), gelatin-capping AgNPs had good potential as anti-infective therapy for *S. aureus*-induced bacterial keratitis treatment. G-AgNPs exhibited strong antibacterial activity against the keratitis-causing bacteria, *S. aureus*, with their ability to disrupt and damage bacterial membranes. The ability of G-AgNPs as anti-infective therapy was also supported by antiangiogenic activity. Moreover, G-AgNPs were bioadhesive and biocompatible, so they could be a promising therapy for microbial eye infections [81].

### 2.2. Anticancer Therapy

Cancer is the major cause of mortality worldwide, being responsible for almost 10 million deaths in 2020 [82]. The challenge in cancer therapy is the dangerous side effects of conventional therapy, which can damage not only cancer cells, but also normal cells [83]. In addition, the use of cancer drugs that exist today is also considered ineffective due to several limitations, such as high toxicity, high cost, susceptibility to resistance, and lack of specificity [84,85]. Therefore, alternative cancer therapies need to be developed, one of which is by using metal nanoparticles. Nowadays, AuNPs and AgNPs have been widely studied due to their low toxicity to normal cells compared to other metal nanoparticles [7]. Their anticancer properties have been tested in vitro toward various types of cancer cells, such as breast cancer [70,71], lung cancer [64,76], liver cancer [66,86], cervical cancer [64], colon cancer [62,87], and human neuroblastoma cells [86]. Several studies have proven that plant-based AuNPs and AgNPs have anticancer biological activity, as shown in Table 3.

AuNPs and AgNPs produced through green synthesis are able to reduce the number of progressive cancer cells with their highly cytotoxic effect on cancer cells. For example, research conducted by Godipurge et al. [70] has shown a decrease in the number of tested cancer cells in the presence of AuNPs and AgNPs synthesized through green synthesis using the *Rivea hypocrateriformis* extract, observed through a decrease in cell viability: the viability of the tested cancer cells (Sf9 and MCF7) decreased to <50%, while normal cells were reduced to 90% at low concentrations (25 μg/mL), and the cell viability decreased as the NP concentration increased. This cytotoxic effect is due to the nanoparticles being highly attracted to biological macromolecules and easily permeating the cell barrier [76,91]. This anticancer activity can occur through several mechanisms, such as apoptosis of cancer cells caused by reactive oxygen species (ROS) which cause damage to cellular components by intracellular oxidative stress, causing cell death [92,93]. The radical scavenging activity also synergistically plays a role in the anticancer activity of AgNPs [94]. The radical scavenging activity is thought to correlate positively with the cytotoxic effect, in which enrichment-free radical scavenging indicates an increased cytotoxic effect [95]. In addition, the anticancer effect of AgNPs could also be caused by reducing the function of mitochondria, the release of lactate dehydrogenase, chromosomal aberrations and DNA damage, induction of gene apoptosis, deregulation of the cell cycle, and the formation of micronuclei [96,97,98]. Meanwhile, AuNPs can work as anticancer agents in several ways, namely in drug delivery, due to their photodynamic and photothermal properties, and as antiangiogenics by inhibiting signalling and normal cell processes due to the binding of VEGF to VEGFR, as well as blocking the phosphorylation of downstream molecules (ERK 1/2, Akt) [99,100]. These AuNPs have specific effects on target cancer cells to push cells toward apoptosis. AuNPs enter cells and target tumour-suppressant genes and oncogenes to stimulate the effective expression of caspase-9, thus encouraging the occurrence of apoptosis [101]. Furthermore, apoptosis can also occur due to the cessation of the cell cycle and inhibition of cytokinesis in AuNPs targeting the nucleus [102].

### 2.3. Anti-Inflammation Agents

Inflammation is the organism’s response as a defence of animal cells against microbial infections or certain injuries [103,104]. The inflammatory response is an important protective reaction against irritation, injury, and infection so that the immune system homeostasis can be maintained. Inflammation can lead to the pathogenesis of various diseases such as autoimmune disorders, rheumatoid arthritis, vascular disease, neurodegenerative disorders, and cancer [104,105,106]. Currently, many synthetic anti-inflammation drugs have had tremendous success. However, their continued use can cause miscellaneous and objectionable side effects [107,108]. In recent decades, nanoparticles have been identified as promising anti-inflammation agents. Nanoparticles have several advantages as anti-inflammation agents, namely having a large surface-area-to-volume ratio, which allows them to be better at blocking inflammatory mediators such as cytokines and inflammation assisting enzymes, better at penetrating inflammatory and epithelial cells so that their effectiveness is better, and better at target selectivity [109,110]. Plant-based AuNPs and AgNPs that play a role as anti-inflammation agents are summarized in Table 4.

Based on the research of Filip et al. [111], AuNPs and AgNPs produced through green synthesis using Cornelian cherry fruit extract, regulate the inflammatory process in the same way as paw tissue injected with carrageenan. The resulting nanoparticles can reduce inflammation and apoptosis in the early stages, then, after 48 h, will provide an immunomodulatory effect and activation of ERK 1/2, and induce the occurrence of apoptosis. These nanoparticles also mobilize antioxidant defence mechanisms against ROS in a short period of time. They also show a modulating effect on the secretion of biphasic pro- and anti-inflammatory cytokines, namely a decrease in the levels of pro-inflammatory cytokines (MCP-1, IL-1α, and IL-1β) and an increase in IL-2 secretion, especially after 24 h and 48 h. Other studies have proven that AuNPs and AgNPs produced using *Prunus serrulata* fruit extract are effective anti-inflammation agents based on in vitro testing of RAW264.7 cells. They can inhibit the activation of NF-κB, thereby increasing the production of pro-inflammatory cytokines and inflammatory mediators [112]. In addition, the biosynthesis of AuNPs and AgNPs using the *Mentha longifolia* extract produces nanoparticles that are able to inhibit cyclooxygenase enzymes (COX-1, COX-2) so that they have painkilling properties as well. These studies proved that AuNPs and AgNPs synthesized using plant extracts are potential alternative agents to treat inflammation [73].

### 2.4. Catalysts

One of the most important uses of metal nanoparticles is as catalysts for some difficult or very slow reactions [67,113], such as reduction reactions for compounds such as 4-nitrophenol, o- and p-nitroaniline, and dyes (rhodamine B, methylene blue, methyl orange, methyl red) using NaBH_4_ [68,75,76,114]. These compounds are chemicals that are dangerous and toxic to living organisms, so the proper degradation of them is required. They are also highly stable in water so that conventional water handling methods usually become ineffective. Therefore, it is important to develop more efficient, cost-effective, and environmentally friendly methods to degrade them using nanocatalysts resulting from the green synthesis process [115,116,117]. In fact, the reduction reaction of these pollutant compounds is thermodynamically permissible, but it is practically and kinetically prohibited even for a couple of days if using only NaBH_4_ alone [118,119]. Nanocatalysts will accelerate the reduction reaction of these pollutant compounds that are reduced using NaBH_4_ as a mediator, by overcoming the kinetic barrier so that electron transfer occurs from the BH^4−^ donor to the acceptor in these pollutant compounds. The process of electron transfer will reduce these pollutant compounds and then they will degrade completely. The reaction proceeds with pseudo-first-order kinetics [120,121,122,123].

As shown in Table 5, several studies have proven that plant-based AuNPs and AgNPs act as promising catalysts in degrading dangerous synthetic chemical compounds in the presence of NaBH_4_ reductors. For instance, the research conducted by Bonigala et al. [114] showed a decrease in compound levels to near zero in the reduction reaction of 4-nitrophenol, methyl red, methyl orange, and methylene blue in the presence of *Stemona tuberosa* extract-based AuNPs and AgNPs. During the process, the success of the reaction can also be seen from the gradual change in the colour of the solution that becomes colourless, for example, for the reaction of 4-nitrophenol, the greenish-yellow colour will disappear slowly as the reaction progresses. The reduction reaction proceeds very rapidly in intervals of just a few minutes even with the addition of a small amount of Au and Ag nanocatalysts when compared to the reaction where no catalyst is added in which, based on testing after a few hours, the compound does not undergo any changes judging from the colour change [68,117,124]. Another study conducted on the reaction reducing o- and p-nitroaniline to 1,2-benzene diamine showed a faster complete reduction reaction, requiring 10 min using AgNP catalyst–*Indigofera tinctoria* extract and 18 min using AuNP catalyst–*Indigofera tinctoria* extract [75]. The reaction rate using AgNPs is attributed to their small size and spherical shape, which allows for the availability of a large number of binding sites for reactant chemisorption that can increase the reaction rate. Meanwhile, the AuNPs have a distinct morphology and are anisotropic, so that they impact the reaction rate [75,125].

Metal nanoparticles can also play a role as catalysts in oxidation reactions such as oxidation reactions of alcohols, alkenes, cyclohexane, and toluene [126,127]. However, plant extract-based AuNPs and AgNPs as oxidation catalysts have not been widely explored. Musere and colleagues reported that AgNPs produced using *Pennisetum glaucum* husk extract could be used as efficient oxidation catalysts. It was able to convert 90% benzyl alcohol to benzaldehyde within 4 h at 80 °C. This result was more efficient when compared to previous study by [128], which took 6 h at 111 °C to convert benzyl alcohol substituted AgNO_3_ and Na_2_CO_3_. The mechanism was thought to be a AgNP-facilitated reaction between benzyl alcohol and -OH, whereby radicals from hydrogen peroxide reduce the bond dissociation energy, increasing the efficiency of electron transfer. Hereinafter, this catalyst was removed after the reaction was completed. It was due to the heterogeneous characteristic of the AgNP catalyst [129].

### 2.5. Drug Delivery Vehicles

Nanoparticles are widely utilized as drug delivery vehicles for a broad range of therapeutic agents, such as cancer therapies, antibodies, peptides, etc. [130,131]. Nanoparticles of various shapes and sizes are currently being proposed to be used as cancer therapies, with the goal of reducing the rate of release and the number of drug doses required [132,133]. Nanoparticles such as liposomes, quantum dots, dendrimers, polymers, and metal nanoparticles, have attracted a lot of attention in medicine. Nowadays, metal nanoparticles, in particular, are being investigated for their significant medical potential to be conjugated into drug delivery vehicles. The materials most investigated for use as nanoparticles in various medicinal areas are gold, silver, and platinum [100,134,135,136]. Among the three, AuNPs have the greatest potential due to their remarkable properties, which make them the ideal drug delivery vehicles [137,138]. AuNPs are simple to prepare, inert, biocompatible, non-cytotoxic, exhibit high tissue permeability, and are stable, so they are suitable for use as nanocarriers [88,137,139]. The structural design of the resulting AuNPs allows for coating of the surface with a variety of targeting agents. Conjugating drugs with AuNPs will minimize side effects because targeted AuNPs will specifically interact with cancer cells and increase the accumulation of intracellular drugs so that the cytotoxic properties of the drug will increase. AuNPs can also be used as target carriers in cancer diagnosis [138]. Table 6 presents information about plant-based AuNPs and AgNPs that act as drug delivery vehicles.

Park Ji Su and colleagues have shown that AuNPs produced through green synthesis using *Garcinia mangostana* (GM) pericarp extract are effective drug delivery vehicles for diagnostic and therapeutic use. GM-AuNPs have weak cytotoxic properties in both normal cells and NIH3T3 and A549 cancer cells. They also have good biocompatibility seen from the morphology of GM-AuNP-treated cells which exhibit no significant changes when compared to carrier controls [88]. Moreover, AuNPs and AgNPs green-synthesized using *Dendropanax morbifera* (DM) leaf extract have been proved to have a synergistic effect in increasing the cytotoxicity and cell apoptosis induction properties of ginsenoside compound K as a natural product for cancer therapy. However, DM-AgNPs exhibit strong cytotoxicity in A549 lung cancer cells, while DM-AuNPs have no cytotoxic effect on either cell type. Because of their non-cytotoxic properties in normal cells, these results suggest that DM-AuNPs can be used as carriers in drug delivery cancer therapy [90]. Other studies have also shown that AuNPs generated by green synthesis with *Cibotium barometz* root extract and *Angelica pubescens* Maxim root extract have the potential to be drug delivery carriers due to their non-toxic properties [77,140]. However, based on these studies, there is no further explanation regarding the type of drugs that can be delivered using plant-based AuNPs as carriers.

### 2.6. Colorimetric Detectors

AuNPs and AgNPs are currently used as colorimetric detectors for metal ions (Cd, Hg, Fe, etc.) [141,142]. Their application has emerged as one of the most promising analytical approaches for detecting analytes. These kinds of colorimetric detectors have numerous advantages, including high sensitivity, ease of measurement, fast metal ion tracking, on-site monitoring, and cost-effectiveness. The general mechanism is the presence of discoloration that can be detected with the naked eye. The spectrophotometry results also support the properties of AuNPs/AgNPs as colorimetric detectors, namely by the presence of red and blue shifts in absorption, which result from the aggregation and disaggregation of nanoparticles [143,144,145]. Table 7 provides a summary of plant-based AuNPs and AgNPs as colorimetric detectors.

Based on their research, Zayed et al. [146] reported that AgNPs produced through green synthesis using *Ficus retusa* leaf ethanol extract are selective and sensitive colorimetric sensors of Fe^3+^ ions in water. Visual inspection shows that the selective addition of Fe^3+^ immediately alters the yellow colour of the AgNP solution into an almost colourless one. Furthermore, the addition of Fe^3+^ ions to the AgNP solution causes the intensity of the SPR band to slowly reduce, with band broadening and blue shift from 426 to 401 nm. This suggests that the addition of Fe^3+^ ions reduces the size and amount of AgNPs in the solution significantly. The detection mechanism of Fe^3+^ ions using *F. retusa*-stabilized AgNPs is an oxidation–reduction reaction between AgNPs and Fe^3+^ ions, which causes the AgNPs to decompose into Ag^+^ ions.

## 3. Phytochemical Content of Plants Used for Gold and Silver Nanoparticle Synthesis

**Figure 2:**
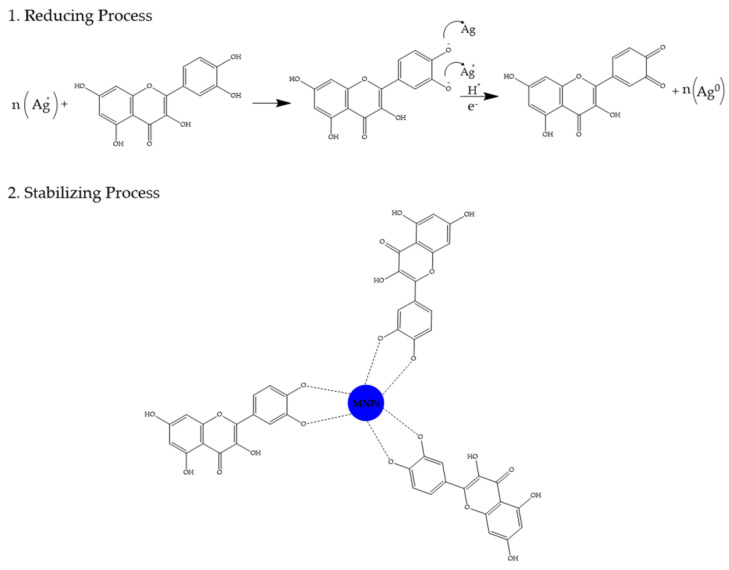
Schema of reducing and stabilizing NPs utilizing polyphenols (e.g., the flavonoid quercetin).

Plants contain phytochemical compounds in their extracts that act as reducing agents and/or natural stabilizers in the biosynthesis of AuNPs and AgNPs [57]. The utilization of phytochemical compounds in the biosynthesis of AuNPs and AgNPs, along with the characteristics of the resulting nanoparticles has been selectively summarized and is shown in Table 8. Plant extracts contain many active phytochemical compounds, such as polyphenols (phenolic acids, flavonoids, lignans, tannins), reducing sugars, polysaccharides, glycosides, alkaloids, triterpenoidal saponins, proteins, steroids, triterpenoids, saponins, fatty acids, and organic acids. Research about the biosynthesis of AuNPs and AgNPs utilizing plant extracts has shown that the polyphenol class of flavonoids can reduce gold and silver ions into nanoparticles and stabilize them. A schema of reducing and stabilizing nanoparticles by utilizing polyphenols can be seen in Figure 2 [57,71,77,89,140]. The flavonoids provide electrons in the reaction of reducing metal ions to nanoparticles through the de-bonding of -O-H bonds from their enol form. Based on FTIR spectrum analysis, it is known that there is a shift in the wave number towards a higher value for both types of nanoparticles compared to the extract, as well as a shift of the C=O group in a lower direction which indicates that flavonoids are also present on the surface of AuNPs and AgNPs, and that oxidation of the flavonoid hydroxyl group to the ketonic group occurs. These flavonoids may react with metal ions through the hydroxyl group of the catechol part, of which the dissociation energy is lower than that of the hydroxyl group on the aromatic ring, and reduce metal ions to nanoparticles. Furthermore, there is a nanoparticle stabilization process, where the enol form of flavonoids that are oxidized into a keto form as a result of compensation from the reaction of reducing metal ions to nanoparticles will stick to the surface of the nanoparticles to prevent their aggregation [57,152,153].

In addition, other studies report that reducing sugars can also play a role in the production of AuNPs and AgNPs, as well as stabilizing the resulting nanoparticles [39,61]. Reducing sugars between polysaccharides play a role in the reduction process by transferring electrons to metal ions and undergoing oxidation of aldehyde groups into carboxylic acids in the synthesis of nanoparticles. The process of stabilizing nanoparticles is assisted by other phytochemical compounds, such as flavonoids and proteins [64,154].

Besides reducing sugars, many proteins are reported to act as capping mediators in the synthesis of plant-based AuNPs and AgNPs [62,67,72,112,155]. Proteins can stabilize nanoparticles through cysteine residues or free amino groups in proteins. Proteins can also be bound to nanoparticles through carboxylic ions free of amino acid residues that have hydroxyl functional groups [67,156]. Based on the research of Sheny et al. [155], in the synthesis of AuNPs using *Anacardium occidentale* protein extract, aliphatic amines and gallic acid act as reducing and capping agents. Meanwhile, in the production of AgNPs, the reduction process is carried out by gallic acid which is also bound to the surface of AgNPs as a capping agent with proteins (glutamic acid, leucine). These proteins will prevent the agglomeration of nanoparticles and stabilize them [72].

Based on the reported studies, polyphenol compounds, reducing sugars, and proteins are the main reducing and stabilizing agents used in the biosynthesis of nanoparticles. This is in line with the previous literature, which states that these three components are the main reducing and stabilizing agents in the synthesis of nanoparticles utilizing plant extracts [161].

## 4. Physical Properties of Nanoparticles Produced Using Plant Extracts

The concentration of extract used can affect the size and shape of the resulting nanoparticles. It takes the optimum concentration to produce nanoparticles with ideal physical properties. Before reaching the optimum concentration, the higher the concentration of the extract used, the smaller the size of the nanoparticles formed. This is due to the increasing concentration of extracts being in line with the increasing number of phytochemical compounds contained in them that act as reducers and stabilizers in the biosynthesis process of AuNPs and AgNPs, so that the rate of nanoparticle formation reaction increased with a smaller size compensation [162,163]. For example, two of the studies shown in Table 8 analysed the content of the main phytochemical compounds in the form of polyphenol compounds (measured as gallic acid) and reducing sugars (measured as glucose). Using 0.403 mg/g polyphenol compounds and 5.02 mg/g reducing sugars, 5–10 nm AuNPs and 10–20 nm AgNPs were produced. Meanwhile, larger particles were produced at lower concentrations of polyphenol compounds and reducing sugars (0.241 and 4.5 mg/g, respectively), namely AuNPs with an average size of 189 nm and AgNPs with an average size of 126 nm. Based on these results, it can be concluded that increasing the content of phytochemical compounds in plant extracts can generate smaller nanoparticles [39,61].

However, when the optimum extract concentration is reached, the contrary will happen because the metal ion bio-reduction process occurs in a saturated state produced due to the saturation of electron injection from plant extracts into nanoparticles [162,163]. The effect of plant extract concentration on the size of nanoparticles is presented in Table 9. Sheny et al. [155] reported that the concentration of *Anacardium occidentale* extracts used to produce metal nanoparticles shows a linear relationship with particle size. In *Anacardium*-based AuNPs and AgNPs, an increase in the quantity of extract added will make the resulting nanoparticles larger as the resulting absorbance tends to increase, and there is a change in the colour of the solution from wine-red to violet. Moreover, the biosynthesis of AuNPs and AgNPs utilizing Siberian ginseng extract with a concentration ratio between metal ions and extracts of 1:1, 1:2, and 1:3, proved that a 1:1 extract ratio provides the best results in the synthesis of both types of nanoparticles [39]. The linear relationship between extract concentration and nanoparticle size was also proven by research conducted by Jiménez et al. [61] related to the biosynthesis of AuNPs and AgNPs using ginseng berry extract (1–8% *v*/*v*), the optimal extract concentration for this biosynthesis process being 5%. When the extract concentration was increased to 6%, the results showed an increase in the wavelength at that concentration. These results state the possibility of an increase in particle size. The higher the concentration of the extract, the more reducing and stabilizing agents will be introduced into the reaction mixture, resulting in a secondary reduction process on the surface of the preformed nucleus. In addition, the presence of additional interactions between surface biomolecule can produce larger particles [61,162,164].

Furthermore, the morphology of the resulting nanoparticles is also influenced by the concentration of the plant extract used and the levels of phytochemical compounds in the extract. Based on research conducted by Jiménez et al. [61] and Abbai et al. [39], it can be concluded that the higher the levels of phytochemical compounds in the extract, the more likely the production of nanoparticles with a regular form, namely spherical, as can be seen in Table 8. Spherical nanoparticles that have a minimum surface-to-volume ratio are more thermodynamically stable. Thus, if the reduction of metal ions takes place under controlled thermodynamic conditions, the main nanoparticles produced are spherical [166]. The previous statement is supported by the biosynthetic study of AuNPs and AgNPs reported by Ahmad et al. [87] using *Trapa natans* extract with phenolic compounds (gallic acid and quinones), which showed a change in the shape of nanoparticles to a tendency to be uniformly spherical (a regular form of nanoparticles) at high concentrations from varying shapes (spherical, hexagonal, triangular) at low concentrations.

Related to the potential activity of nanoparticles, a higher concentration of the possible extract will indicate greater activity as well. The resulting morphology affects the activity of plant-based nanoparticles. A well-tuned morphology tends to increase the surface area of the nanoparticle, increasing its active site and directly affecting its activity [167]. For instance, in *Pulicaria undulata*-based nanoparticles, it has been proven that a higher concentration of extract results in improved catalytic activity against the reduction of 4-nitrophenol due to the formation of smaller nanoparticles with well-defined spherical morphology. Meanwhile, inefficient catalytic activity is demonstrated by ill-defined irregular-shaped nanoparticles formed with low extract concentrations [60]. In addition, the size of plant-based nanoparticles also has an essential influence on their activity, such as catalytic activity: in the reduction reaction of fenugreek-based AuNPs, a decrease in the size of nanoparticles causes an increase in the reduction rate. This illustrates that the catalytic activity of nanoparticles increases due to a rise in the number of poorly coordinated Au atoms that promote the reactant adsorption on the surface of the catalyst and allows for reduction [165]. Besides the activity of the extracts, their concentration also affects the stability of the nanoparticles due to an increase in the adsorption of phytochemical compounds that act as stabilizers on the surface of nanoparticles [40].

**Figure 3:**
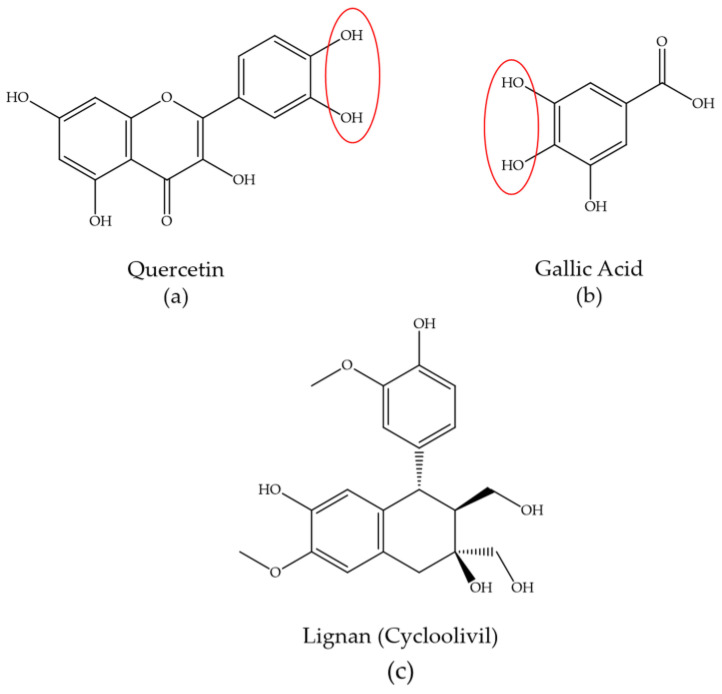
Structure of polyphenolic compounds contained in plant extracts. (**a**,**b**) Polyphenols with ortho-substituted hydroxyl. (**c**) Polyphenol without ortho-substituted hydroxyl. Note: red circle is ortho substituted hydroxyl group as preferred complexation sites.

Considering the structure of phytochemical compounds contained in the plant extracts, the number of hydrogen ions in the hydroxyl group possessed by a phytochemical compound will affect the size and shape of the nanoparticles formed [168,169]. In the process of metal nanoparticle synthesis, a reduction–oxidation reaction occurs in which phytochemical compounds in the plant extract act as reducers to reduce metal ions to metal atoms. The hydroxyl groups will react with metal ions by transferring electrons from the protonated hydroxyl group to metal ions, so that a reduction process occurs. The hydroxyl groups will initially bind metal ions to form chelating, rings which will oxidize into carbonyl-based compounds with the change of metal ions into atoms. In this process, the resulting atoms will form aggregates that come together to form small nanoparticles. Furthermore, these nanoparticles will grow autocatalytically and increase in size. However, after forming particles of an appropriate size, the hydroxyl groups oxidized to the carbonyl groups will form a steric layer on the surface of the nanoparticles to stop aggregation and stabilize it [153,168,170]. The chelating process occurs preferably in ortho-substituted hydroxyl groups. For example, the gallic acid chelating site in the metal ion reduction reaction is indicated at the ortho-substituted hydroxyl position. In addition, in flavonoids, the preferred complexation sites are the OH group on carbons 3 or 5 and the adjacent 4-carbonyl group. This is possibly due to its bond dissociation energy being lower than that of the hydroxyl groups at other positions [152,153,171,172]. Based on Table 8, when examining plant-based nanoparticles produced utilizing polyphenols, using quercetin and gallic acid compounds that have an ortho-substituted hydroxyl, as seen in Figure 3, results in an average nanoparticle size that is smaller than when using lignans, even though all of the nanoparticles have the same regular spherical shape [57,69,87]. Besides being influenced by the concentration and amount of phytochemical compounds in the plant extracts, green synthesis of AuNPs and AgNPs is also influenced by several factors, such as metal ion concentration, temperature, reaction time, and pH [173]. In each plant-based biosynthesis process of AuNPs and AgNPs, optimal conditions of each factor are required for the successful synthesis of nanoparticles, as shown in Table 8.

The concentration of metal ions plays an important role in the biosynthesis of AuNPs and AgNPs. The produced nanoparticle size becomes larger as the concentration of metal ions increases. This is due to bio-reduction in the nanoparticle synthesis reactions decreasing as the concentration of metal ions increases [157]. In addition, when the concentration of metal ions exceeds the optimal amount, polydispersion nanoparticles will form. This is likely due to the lower capping effect of the extract at higher concentrations of metal ions [146]. In a study conducted by Devi and Sathishkumar [89], optimization of the effect of metal ion concentration on the synthesis of AuNPs and AgNPs utilizing *Mukia maderaspatna* extract was tested. This study used various concentrations of metal ions, with a fixed temperature and the amount of plant extracts. In the synthesis of AuNPs, the concentration of HAuCl_4_ varied from 0.1 to 5 mM, with a fixed temperature of 80 °C. The optimal conditions for the synthesis of AuNPs were found at a concentration of 2 mM, with a maximum absorbance at 531 nm. There was peak broadening as the concentration of metal ions increases. This is due to an increase in nanoparticle size. Meanwhile, in the synthesis of AgNPs, the concentration of AgNO3 varied from 0.1 to 1 mM, with a fixed temperature of 70 °C. Maximum absorbance was obtained at a concentration 1 mM at a wavelength of 431 nm and a wavelength shift at a greater concentration.

Temperature also plays a role in the biosynthesis process of AuNPs and AgNPs, in which an increase in reaction temperature can increase the yield of AuNPs and AgNPs [174]. An increase in temperature can lead to an increase in the sharpness of the peak with wavelength shifts. This may be due to the aggregation of growth nanoparticles decreasing with increasing temperature [175]. In addition, an increase in temperature accelerates the reduction process. As reported in the research about the green synthesis of AuNPs and AgNPs utilizing the *Zingiber officinale* extract, the absorbance of the reaction mixture increases with increasing temperature from 20 to 50 °C [74]. Jimenez et al. [61] reported that as the reaction temperature raises, the reaction time decreases and the particle size of AuNPs and AgNPs gets smaller. Nanoparticle synthesis utilizing ginseng berry extract performed at a low temperature (23 °C) showed slow reduction reaction rates and the total reaction times for AuNPs of 270 min and 24 h, as well as surface plasmon wavelengths of AuNPs and AgNPs at 540 and 444 nm, indicating larger particle sizes compared to synthesis at 90 °C. When the reaction temperature was raised to 90 °C, the maximum absorption occurs at wavelengths of 530 and 422 nm for AuNPs and AgNPs. Furthermore, there was a significant increase in the reaction speed, with total reaction times for AuNPs at 25 min and AgNPs at 3 h.

Reaction time is an important parameter which increases in absorbance, in line with the increase in incubation time. The synthesis of AuNPs using *Mukia maderaspatna* extract showed that in the first 30 min of incubation time, there was no formation of nanoparticles. The metal ion reduction process began after 1 h of incubation, which was indicated by an increase in absorbance of up to 4 h of incubation time. However, further observations until the reaction time reached 21 h showed a change in the maximum wavelength from 538 nm to 531 nm. It could be concluded that the synthesis of AuNPs had been completed at 4 h incubation time. Meanwhile, in the synthesis of AgNPs, the nanoparticle formation reaction began rapidly after 10 min of incubation time at a maximum wavelength of 424 nm. The absorbance increased with increasing incubation time without a shift in maximum wavelength. The optimum incubation time for the synthesis of AgNPs was 30 min. Incubation after optimal conditions resulted in the aggregation of AgNPs, leading to an increase in nanoparticle size [89].

Several studies have proven that pH has an important role in the synthesis of AuNPs and AgNPs. Studies conducted by Zayed et al. [146] and Velmurugan et al. (2014) reported that the optimal conditions for nanoparticle synthesis were carried out at an alkaline pH. AgNPs synthesized using the *Ficus rectusa* extract showed increased intensity, narrowing of width, and blueshift from the SPR peak in the pH range of 6–9, indicating a decrease and diversity in size within that pH range. The optimal pH of AgNPs synthesis was 9. At pH 9, the prepared AgNPs exhibited a spherical shape, absence of agglomeration, and narrowness of particle size distribution. Meanwhile, AuNPs synthesized using the *Ficus rectusa* extract also showed good results in the pH range of 6–9, with an optimal pH value of 6. At pH 6, the formed AuNPs were well dispersed in the absence of aggregation, with a size range of 10–25 nm and quasi-spherical particle shape. Meanwhile, AuNPs made at pH 2 showed more diverse nanoparticle shapes, namely spherical and irregular triangular, with a particle size range of 5–20 nm. Velmurugan et al. [74] also reported that there was an increase in absorbance when increasing pH from 4 to 9. The optimal pH for the formation of AgNPs and AuNPs was pH 8 and pH 9, which was pH alkaline. This was due to the alkaline media having many hydroxyl ions that could accelerate the transfer of electrons from phytochemical compounds in extracts to metal ions [176]. Biosynthesis of AuNPs and AgNPs carried out on this alkaline medium could shorten the nucleation stage and lead the reaction into the growth stage [177].

## 5. Conclusions

This review was written to gain insight into the synthesis of AuNPs and AgNPs utilizing the phytochemical compounds in plants, the properties of nanoparticles based on the concentration and structure of phytochemical compounds, as well as their application. The biosynthesis process needs to establish the optimal conditions, such as the concentration of the plant extract/phytochemical contents, metal ion concentration, temperature, reaction time, and pH, to get the good characteristics of formed nanoparticles. Based on several studies that have been discussed in this article, it can be concluded that phytochemical compounds play an essential role in the biosynthesis of AuNPs and AgNPs as reducing agents and stabilizers. The main phytochemical compounds that play an important role in the biosynthesis of AuNPs and AgNPs are polyphenols, reducing sugars, and proteins. With the knowledge about the phytochemical compounds that play a role in the biosynthesis of AuNPs and AgNPs, it is possible to control the physical properties of the formed nanoparticles. The higher level of phytochemical content in the extract leads to produce nanoparticles with regular shapes and smaller sizes. Furthermore, considering the structure, the phytochemical compounds that have ortho-substituted hydroxyl also result in a smaller size and well-defined shape. These conditions can lead to a greater activity and stability of the formed nanoparticles. However, most of the studies reviewed in this article have not performed analyses to determine the specific phytochemical contents and their concentrations contained in the extracts, such as HPLC analysis. Therefore, further studies are needed given the importance of knowledge regarding the chemical compounds in the extracts used in the biosynthesis of AuNPs and AgNPs.

AuNPs and AgNPs can be utilized for their potential activities in various fields, both medical, such as antimicrobial and antibiofilm agents, anticancer therapy, anti-inflammation agents, and non-medical, including catalysts in the reduction and oxidation reactions, colorimetric detectors, and drug delivery vehicles. In the medical field, the exploration of in vivo studies is still needed. Meanwhile, in the non-medical field, especially regarding drug delivery vehicles, we found a lack of sources that explained the types of drugs that can be carried by nanoparticles. Therefore, further studies need to be carried out by researchers to evaluate the application of nanoparticles as drug-delivery vehicles of several types of drugs.

## Figures and Tables

**Table 1:** Advantages and disadvantages of green synthesis of NPs using plant extracts and microorganisms.

Method	Advantages	Disadvantages
Plant-based biosynthesis	High speed, eco-friendly, pollutant- and toxicity-free, more cost-effective (no cost for culture media and microorganism isolation), simple handling, stable and non-aggregated NPs, scalability	Cannot be genetically manipulated like microorganisms
Microorganism-based biosynthesis	Eco-friendly, non-toxic, clean, can be manipulated easily	Low speed, complicated process (sampling, isolation, culturing, storage of microorganisms, and downstream processing), difficult to control stability and aggregation, cost- and time-consuming process (need to culture microorganisms), probability of endotoxin presence

**Table 2:** Plant-based gold and silver nanoparticles as antimicrobial and anti-biofilm agents.

Plant	Part of the Plant	Target Pathogens	Activity	Concentration	Type of NP	Experimental Outcomes	Ref.
*Amorphophallus paeoniifolius*	Tuber	*Pseudomonas aeruginosa*, *Escherichia coli*, *Salmonella typhimurium*, *Citrobacter freundii*, *Bacillus subtilis*, *Staphylococcus aureus*	Antibacterial	25 µL/disk	Ag	The highest activity against *P. aeruginosa* with the ZOI was 20 nm at a concentration of 25 µL/disk	[57]
					Au	Did not show activity (ZOI = 0.00 nm)	
*Gloriosa superba*	Leaf	*Bacillus subtilis*, *Escherichia coli*	Antibacterial	10–50 µL/disk	Ag	The highest activity against *E. coli* with the ZOI was 7.66 ± 0.33 mm at a concentration of 30 µL/disk	[58]
					Au	Did not show activity (ZOI = 0.00 nm)	
*Rhodiola rosea*	Rhizo-me	*Pseudomonas aeruginosa*, *Escherichia coli*	Antibacterial	50–200 µg/mL	Ag	Significant activity against *P. aeruginosa* with an MIC and MBC at 50 µg/mL and 100 µg/mL; meanwhile, the MIC and MBC against *E*. *coli* were 100 µg/mL and 200 µg/mL	[59]
					Au	Did not show activity	
			Antibiofilm	1.6–200 µg/mL	Ag	Significant activity was at a concentration of ≥ 6.25 µg/mL	
					Au	Significant activity was at a concentration of ≥ 12.5 µg/mL	
*Clerodendrum* *inerme*	Leaf	*Staphylococcus aureus*, *Bacillus subtilis*, *Klebsiella*, *Escherichia coli*	Antibacterial	250 µg/mL	Ag	The highest activity against *Klebsiella* with the ZOI was at 21 µg/mL	[60]
					Au	The highest activity against *E. coli* with the ZOI was at 16 µg/mL	
		*Aspergillus niger*, *Aspergillus flavus*, *Trichoderma harzianum*	Antimycotic	250 µg/mL	Ag	The highest activity against *A. flavus* with the ZOI was at 22 µg/mL	
					Au	the highest activity against *A. flavus* with the ZOI was at 20 µg/mL	
*Panax ginseng* Meyer	Fruit	*Escherichia coli*, *Staphylococcus aureus*	Antibacterial	1.15–3.45 µg/disk	Ag	The highest activity against *S. aureus* with the ZOI was 12.3 mm at a concentration of 3.45 µg/disk	[61]
					Au	-	
Siberian ginseng (*Eleutherococcus senticosus*)	Stem	*Escherichia coli*, *Vibrio parahaemolyticus*, *Staphylococcus aureus*, *Bacillus anthracis*	Antibacterial	10–30 µg/mL	Ag	The highest activity against *S. aureus* with the ZOI was 13.8 ± 0.2 mm at a concentration of 30 µg/mL	[39]
					Au	Did not show activity (ZOI = 0.00 nm)	
*Actinidia deliciosa*	Fruit	*Pseudomonas aeruginosa*	Antibacterial	10–30 µg/mL	Ag, Au	AgNPs showed a higher ZOI than AuNPs at each concentration	[62]
*Rosa canina* L.	Rose-hip	*Escherichia coli*	Antibacterial	0.5 µg/mL	Ag	MIC was 0.5 µg/mL	[63]
					Au	-	
*Borago officinalis*	Leaf	*Pseudomonas aeruginosa*, *Vibrio parahaemolyticus*, *Staphylococcus aureus*, *Escherichia coli*	Antibacterial	15 µL/disk	Ag	The highest activity against *P. aeruginosa* with the ZOI was 13.7 ± 0.5 mm at a concentration of 15 µL/disk	[64]
			Antibiofilm	2–10 µg/mL	Ag	Significant activity was at a concentration of 10 µg/mL	
*Asparagus racemosus*	Root	*Pseudomonas aeruginosa*, *Staphylococcus aureus*, *Escherichia coli*, *Bacillus subtilis*, *Klebsiella pneumonia*	Antibacterial	20–80 µg/mL	Ag	The highest activity against *P. aeruginosa* with the ZOI was 28 mm at a concentration of 80 µg/mL	[65]
					Au	The highest activity against *P. aeruginosa* with the ZOI was 26 mm at a concentration of 80 µg/mL	
*Coleus forskohlii*	Root	*Proteus vulgaris*, *Micrococcus luteus*, *Pseudomonas aeruginosa*, *Staphylococcus aureus*, *Escherichia coli*	Antibacterial	15–35 µL/disk	Ag	The highest activity against *E. coli* with the ZOI was 26 mm at a concentration of 35 µL/disk	[66,67]
					Au	The highest activity against *E. coli* with the ZOI was 21 mm at a concentration of 35 µL/disk	
*Mussaenda glabrata*	Leaf	*Pseudomonas aeruginosa*, *Bacillus pumilus*, *Escherichia coli*, *Staphylococcus aureus*,	Antibacterial	-	Ag	The highest activity against *S. aureus* with the ZOI was 19 mm	[68]
					Au	The highest activity against *S. aureus* with the ZOI was 14 mm	
		*Aspergillus niger*, *Penicillium chrysogenum*	Antimycotic	-	Ag	The highest activity against *P. chrysogenum* with the ZOI was 13 mm	
					Au	The highest activity against *P. chrysogenum* with ZOI was 12 mm	
*Stereospermum chelonoides*	Root bark	*Bacillus subtilis*, *Staphylococcus aureus*, *Pseudomonas aeruginosa*, *Escherichia coli*	Antibacterial	1000 µg/mL	Ag	The highest activity against *P. aeruginosa* with the ZOI was 20 mm	[69]
					Au	The highest activity against *E. coli* with the ZOI was 17 mm	
		*Aspergillus flavus*, *Aspergillus nidulans*	Antimycotic	1000 µg/mL	Ag	The highest activity against *A. flavus* with the ZOI was 23 mm	
					Au	The highest activity against *A. flavus* with the ZOI was 21 mm	
*Rivea hypocrateriformis*	Aerial part	*Klebsiella pneumonia*, *Bacillus subtilis*, *Staphylococcus aureus*, *Pseudomonas aeruginosa*, *Escherichia coli*	Antibacterial	25–100 µg/mL	Ag	The highest activity against *E. coli* with the ZOI was 13 mm at a concentration of 100 µg/mL	[70]
					Au	The highest activity against *E. coli* with the ZOI was 12 mm at a concentration of 100 µg/mL	
		*Trichophyton rubrum*, *Candida albicans*, *Chrysosporium indicum*	Antimycotic	25–100 µg/mL	Ag	The highest activity against *C. indicum* with the ZOI was 7 mm at a concentration of100 µg/mL	
					Au	The highest activity against *C. indicum* with the ZOI was 6 mm at a concentration of 100 µg/mL	
*Glycyrrhiza uralensis*	Root	*Pseudomonas aeruginosa*, *Salmonella enterica*, *Staphylococcus aureus*, *Escherichia coli*	Antibacterial	15–45 µg/disk	Ag	The highest activity against *S. aureus* with the ZOI was 17.3 ± 0.57 mm at a concentration of 45 µg/disk	[71]
					Au	Did not show activity	
*Parkia roxburghii*	Leaf	*Escherichia coli*, *Staphylococcus aureus*	Antibacterial	25 µL/disk	Ag, Au	AgNPs showed the higher ZOI than AuNPs at each strain	[72]
*Mentha longifolia*	Leaf	*Staphylococcus aureus*, *Bacillus subtilis*	Antibacterial	3000 µg/mL	Ag	The highest activity against *S. aureus* with the ZOI was 12 ± 0.03 mm	[73]
					Au	The highest activity against *S. aureus* with the ZOI was 10 ± 0.01 mm	
*Zingiber officinale*	Root	*Listeria* spp., *Staphylococcus* spp.	Antibacterial	25 µL/disk	Ag	The highest activity against *Listeria* spp. with the ZOI was 8.9 ± 0.6 mm	[74]
*Indigofera tinctoria*	Leaf	*Staphylococcus aureus*, *Bacillus pumilus*, *Pseudomonas aeruginosa*, *Escherichia coli*	Antibacterial	100 µL/disk	Ag, Au	AgNPs showed the higher ZOI than AuNPs at each strain	[75]
		*Aspergillus fumigatus*, *Aspergillus niger*	Antimycotic	100 µL/disk	Ag, Au	AgNPs showed the higher ZOI than AuNPs at each strain	
*Bauhinia purpurea*	Leaf	*Staphylococcus aureus*, *Bacillus subtilis*, *Pseudomonas aeruginosa*, *Escherichia coli*	Antibacterial	50 µL/disk	Ag, Au	AgNPs showed the higher ZOI than AuNPs at each strain	[76]
		*Aspergillus nidulans*, *Aspergillus flavus*	Antimycotic	50 µL/disk	Ag, Au	AgNPs showed the higher ZOI than AuNPs at each strain	
*Cibotium barometz*	Root	*Staphylococcus aureus*, *Pseudomonas aeruginosa*, *Salmonella enterica*, *Escherichia coli*	Antibacterial	15–45 µL/disk	Ag	The highest activity against *S. aureus* with the ZOI was ± 16 nm	[77]
*Jasminum sambac*	Leaf	*Staphylococcus aureus*, *Pseudomonas aeruginosa*, *Salmonella typhi*, *Escherichia coli*	Antibacterial	10 µg/disk	Ag	The highest activity against *E. coli* with the ZOI was 7 nm	[78]
					Au	The highest activity against *E. coli* with the ZOI was 4 nm	

Abbreviations: ZOI = zone of inhibition; MIC = minimum inhibitory concentration; MBC = minimum bactericidal concentration.

**Table 3:** Plant-based gold and silver nanoparticles as anticancer therapy.

Plant	Part of the Plant	Cell Lines Targeted	Concentration	Type of NP	Experimental Outcomes	Ref.
*Rivea* *hypocrateriformis*	Aerial part	Breast cancer cell (MCF-7)	25 µg/mL	Ag	51.30% cytotoxic effect	[70]
				Au	52% cytotoxic effect	
*Glycyrrhiza* *uralensis*	Root	Breast cancer cell (MCF-7)	10 µg/mL	Ag	20% cytotoxic effect	[71]
			-	Au	Did not show cytotoxic effect	
		Murine macrophage (RAW264.7)	-	Ag	Did not show cytotoxic effect	
			25 µg/mL	Au	15% cytotoxic effect	
*Borago officinalis*	Leaf	Cervical cancer cell (HeLa)	5 µg/mL	Ag	74.90% cytotoxic effect	[64]
		Lung cancer cell (A549)	10 µg/mL	Ag	64.30% cytotoxic effect	
		Murine macrophage (RAW264.7)	2–10 µg/mL	Ag	20–25% cytotoxic effect	
*Indigofera* *tinctoria*	Leaf	Lung cancer cell (A549)	56.62 ± 0.86 µg/mL (IC_50_)	Ag	AgNPs and AuNPs showed more cytotoxic effect than plant extract alone (IC_50_ = 71.92 ± 0.76 µg/mL)	[75]
			59.33 ± 0.57 µg/mL (IC_50_)	Au		
*Bauhinia* *purpurea*	Leaf	Lung cancer cell (A549)	100 µg/mL	Ag	68% cytotoxic effect	[76]
				Au	69% cytotoxic effect	
Rooibos (*Aspalathus linearis*)	Leaf and stem	Human neuroblastoma (SH-SY5Y)	25–500 µg/mL	Ag	Anticancer activity with IC_50_ was 108.80 µg/mL	[86]
				Au	Did not show cytotoxic effect	
		Liver cancer cell (HepG2)	25–500 µg/mL	Ag	Anticancer activity with IC_50_ was 183.40 µg/mL	
				Au	Did not show cytotoxic effect	
*Coleus forskohlii*	Root	Liver cancer cell (HepG2)	10 µg/mL	Ag, Au	20% cytotoxic effect	[66]
Siberian ginseng (*Eleutherococcus senticosus*)	Stem	Breast cancer cell (MCF-7)	10 µg/mL	Ag	40% cytotoxic effect	[39]
			-	Au	Did not show cytotoxic effect	
		Human keratinocyte cell (HaCaT)	10 µg/mL	Ag	17% cytotoxic effect	
			-	Au	Did not show cytotoxic effect	
Mangosteen (*Garcinia* *mangostana*)	Pericarp	Lung cancer cell (A549)	18.75 µg/mL	Ag	11.9% cytotoxic effect	[88]
			75 µg/mL	Au	23.5% cytotoxic effect	
		NIH3T3 cell	37.5 µg/mL	Ag	63% cytotoxic effect	
			75 µg/mL	Au	6.2% cytotoxic effect	
*Actinidia* *deliciosa*	Fruit	Colon cancer cell (HCT116)	350 µg/mL	Ag	22% cytotoxic effect	[62]
				Au	29% cytotoxic effect	
*Mukia* *maderaspatana*	Leaf	Breast cancer cell (MCF-7)	1–100 µg/mL	Ag	Anticancer activity with IC_50_ was 51.30 µg/mL	[89]
				Au	Anticancer activity with IC_50_ was 44.80 µg/mL	
*Dendropanax* *morbifera*	Leaf	Human keratinocyte cell (HaCaT)	100 µg/mL	Ag	40% cytotoxic effect	[90]
				Au	Did not show cytotoxic effect	
		Lung cancer cell (A549)	100 µg/mL	Ag	70% cytotoxic effect	
				Au	Did not show cytotoxic effect	

Note: IC_50_ is the concentration of sample that can cause 50% of the cell death.

**Table 4:** Plant-based gold and silver nanoparticles as anti-inflammation agents.

Plant	Part of the Plant	Type of NP	Experimental Outcome	Ref.
Cornelian cherry (*Cornus mas*)	Fruit	Au, Ag	↓ Inflammation and apoptosis in early stage	[111]
*Prunus serrulata*	Fruit	Au, Ag	↓ Expression of inflammatory mediators in lipopolysaccharide-induced RAW 264.7	[112]
*Mentha longifolia*	Leaf	Au, Ag	The activity of AuNPs and AgNPs (100 mg/kg) was comparable with the standard drug (10 mg/kg)	[73]

Note: ↓ means decreasing effect on pharmacological activity.

**Table 5:** Plant-based gold and silver nanoparticles as catalysts.

Plant	Part of the Plant	Reactions	Type of NP	Experimental Outcomes				Ref.
				↓ Intensity λmax	New Band	Reaction Time	Discoloration	
*Stemona* *tuberosa* Lour	Aerial parts	Reduction of 4-nitrophenol	Au	✓	✓	NE	✓	[114]
			Ag	✓	✓	NE	✓	
		Reduction of methyl orange	Au	✓	✓	NE	✓	
			Ag	✓	✓	NE	✓	
		Reduction of methyl red	Au	✓	✓	NE	✓	
			Ag	✓	✓	NE	✓	
		Reduction of methylene blue	Au	✓	✓	NE	✓	
			Ag	✓	✓	NE	✓	
*Mussaenda* *glabrata*	Leaf	Reduction of 4-nitrophenol	Au	✓	✓	7 min	NE	[68]
			Ag	✓	✓	9 min	NE	
		Reduction of rhodamine B	Au	✓	✓	5 min	NE	
			Ag	✓	✓	9 min	NE	
		Reduction of methyl orange	Au	✓	✓	4 min	NE	
			Ag	✓	✓	7 min	NE	
*Indigofera* *tinctoria*	Leaf	Reduction of o- and p-nitroaniline	Au	✓	✓	18 min	NE	[75]
			Ag	✓	✓	10 min	NE	
*Bauhinia* *purpurea*	Leaf	Reduction of rhodamine B	Au	✓	✓	4 min	✓	[76]
			Ag	✓	✓	6 min	✓	
		Reduction of methylene blue	Au	✓	✓	4 min	✓	
			Ag	✓	✓	6 min	✓	
*Aerva lanata*	Leaf	Reduction of 4-nitrophenol	Au	✓	✓	11 min	✓	[117]
			Ag	✓	✓	13 min	✓	
*Platycodon* *grandiflorum*	Radix	Reduction of 4-nitrophenol	Au	✓	✓	720 s	✓	[124]
*Pulicaria* *undulata*	Aerial part	Reduction of 4-nitrophenol	Au	✓	✓	~2 h	✓	[40]
			Ag	✓	✓	> 10 h	✓	
*Actinidia* *deliciosa*	Fruit	Reduction of methylene blue	Au	✓	✓	14 min	✓	[62]
			Ag	✓	✓	22 min	✓	
*Rosa canina* L.	Rosehip	Reduction of 4-nitrophenol	Au	✓	✓	NE	NE	[63]
*Coleus* *forskohlii*	Root	Reduction of 4-nitrophenol	Ag	✓	✓	24 min	✓	[67]
*Glycyrrhiza* *uralensis*	Root	Reduction of methylene blue	Au, Ag	✓	✓	~60 min	NE	[71]

Abbreviation: NE = not explained, ↓ = decreasing effect, ✓ = effect that mention on the text exist.

**Table 6:** Plant-based gold and silver nanoparticles as drug delivery vehicles.

Plant	Part of the Plant	Type of NP	Experimental Outcomes	Ref.
Mangosteen (*Garcinia mangostana*)	Pericarp	Au	Low cytotoxic effect at the highest concentration (6.2%)	[88]
*Dendropanax morbifera*	Leaf	Au	No cytotoxic effect	[90]
*Cibotium barometz*	Root	Au	No cytotoxic effect	[77]
*Angelica pubescens* Maxim	Root	Au	No cytotoxic effect	[140]

**Table 7:** Plant-based gold and silver nanoparticles as colorimetric detectors.

Plant	Part of the Plant	Type of NP	Ion Target	Ref.
*Ficus retusa*	Leaf	Ag	Fe^3+^	[146]
*Moringa oleifera*	Flower	Ag	Cu^4+^	[147]
*Murraya koenigii*	Leaf	Ag	Hg^2+^	[148]
*Ficus benjamina*	Leaf	Ag	Zn^2+^	[149]
*Cinnamomum tamala*	Leaf	Au	Hg^2+^	[150]
*Momordica charantia*	Fruit	Au	Cd^2+^	[151]

**Table 8:** Plant-based gold and silver nanoparticles and their characteristics.

Plant	Part of the Plant	Phytochemical Contents	Concentration of Phytochemicals	Detection Method	Solvent	Synthesis Condition	Type of NP	Size (nm)	Shape	Ref.
*Panax ginseng* Meyer	Fruit	Ginsenosides	-	HPLC	Water	Add 1 mM HAuCl_4_·3H_2_O to 5% extract incubated at 80 °C for 45 min	Au	5–10	Spherical	[61]
		Polyphenols	0.403 ± 0.03 mg/g (measured as gallic acid)	UV-Vis						
		Reducing sugars	5.02 ± 1.70 mg/g (measured as glucose)			Add 1 mM AgNO_3_ to 5% extract incubated at 80 °C for 3.5 h	Ag	10–20		
		Acidic polysaccharides	0.137 ± 5.70 mg/g							
Siberian ginseng (*Eleutherococcus senticosus*)	Stem	Phenolic compounds	0.241 mg/g (measured as gallic acid)	LC-MS	Water	Add 1 mM HAuCl_4_·3H_2_O to 1:1 diluted extract incubated at room temperature for 9 min	Au	189	Face-centred cubical	[39]
		Reducing sugars	4.5 mg/g (measured as glucose)			Add 1 mM AgNO_3_ to 1:1 diluted extract incubated at 80 °C for 1.5 h	Ag	126		
		Proteins	-							
Cornelian cherry (*Cornus mas*)	Fruit	Polyphenolic compounds	-	UV-Vis	Water	Add 10 mL extract to 30 mL 1 mM HAuCl_4_ irradiated UV light at room temperature for 15 min	Au	5–30	Pseudo-spherical	[111]
						Add 10 mL extract to 30 mL 1 mM AgNO_3_ irradiated UV light at room temperature for 2.5 h	Ag	10–25	Spherical	
*Pulicaria* *undulata*	Aerial part	Phenolic compounds (quercetin, kaempferol, dihydrokaempferol, caffeic acid)	-	-	Water	Add 1 mL HAuCl4·3H2O 1 M to 0.2 mL extract 10 mg/mL stirred at room temperature for 2 h	Au	5–12	Regular shape (spherical); irregular shape (triangular, hexagonal, quasi-elongated plates)	[40]
						Add 1 mL AgNO_3_ 1 M to 0.2 mL extract 10 mg/mL stirred at room temperature for 60 min	Ag			
*Rhodiola rosea*	Rhizome	Flavonoids, polyphenols, terpenoids, polysaccharides, alkaloids, vitamins, amino acids, organic acids	-	FTIR	Water	Add 5 mM HAuCl_4_ to 2:8 diluted extract incubated at room temperature for 4 s	Au	12–18	Irregular shape	[59]
						Add 5 mM AgNO_3_ to 2:8 diluted extract incubated at 90 °C for 10 min	Ag	12–30	Spherical	
Mangosteen (*Garcinia* *mangostana*)	Pericarp	Flavonoids, phenolic compounds, carbohydrates, glycosides	-	Phytochemical screening, FTIR	Methanol	Mix 0.35 M HAuCl_4_·3H_2_O and 0.02% extract vortexed 5 s then incubated at room temperature for 5 h	Au	15.37–44.20	Spherical	[88]
						Mix 0.35 M AgNO_3_ and 0.02% extract vortexed 5 s then incubated at room temperature for 5 h	Ag	13.65–31.08	Asymmetric nano-dumbbell	
*Stemona tuberosa* Lour	Aerial parts	-	-	-	Water	Add 1 mL extract to 9 mL 1 mM HAuCl_4_ incubated at 80 °C for 20 min	Au	20–30	Irregular shape	[114]
						Add 1 mL extract to 9 mL 1 mM AgNO_3_ incubated at 80 °C for 5 min	Ag	10–12	Spherical, irregular shape	
*Actinidia* *deliciosa*	Fruit	Proteins	-	FTIR	-	Add 1 mL extract to 49 mL 1 mM HAuCl_4_ incubated for 2 h	Au	7–20	Spherical	[62]
						Add 10 mL extract to 190 mL 1 mM AgNO_3_ incubated for 2 h	Ag	25–40		
*Rosa canina* L.	Rosehip	Phenolic compounds	-	FTIR	Water	Add 80% diluted extract to 1 mM HAuCl_4_ (1:1) incubated for 15 min	Au	26	Quasi-spherical	[63]
						Add 80% diluted extract to 10 mM AgNO_3_ (1:1) incubated for 15 min	Ag	34		
Rooibos (*Aspalathus* *linearis*)	Leaf and stem	Polyphenols, aspalathin	-	FTIR	Water	Add 10 mL 5% extract to 90 mL 1 mM HAuCl_4_ (heated at 70 °C), stirred under reflux for 28 min	Au	7.5 ± 0.34	Hydra-like shape	[86]
						Add 10 mL 5% extract to 90 mL 1 mM AgNO_3_ (heated at 70 °C), stirred under reflux for 30 min	Ag	6.7 ± 0.39	Quasi-spherical	
*Borago officinalis*	Leaf	Reducing sugars, saccharides, proteins, flavonoids	-	FTIR	Water	Add AgNO_3_ to 25% extract with final concentration 1 mM, incubated at 65 °C for 65 s	Ag	30–80	Spherical, hexagonal, irregular shape	[64]
*Ficus retusa*	Leaf	Phenolic compounds	-	FTIR	Ethanol	Add 100 µL extract to 0.75 mM HAuCl_4_ at pH 6 for 75 min	Au	10–25	Spherical	[146]
						Add 200 µL extract to 1.5 mM AgNO_34_ at pH 9 for 60 min	Ag	15		
*Mukia maderaspatana*	Leaf	Flavonol (quercetin, phloroglucinol)	-	Phytochemical screening	Water	Add 2 mM HAuCl_4_ to 10 mL extract incubated at 80 °C for 4 h	Au	20–50	Spherical, triangular, circular	[89]
						Add 1 mM AgNO_3_ to 10 mL extract incubated at 70 °C for 30 min	Ag	20–50	Irregular shape	
*Clerodendrum inerme*	Leaf	Phenolics, flavonoids, cardiac glycosides, anthraquinones, carbohydrates	-	-	Water	Add 1 mM HAuCl_4_·3H_2_O to 25 mL extract heated at 80 °C for 65 min with continuous stirring	Au	5.82	Spherical	[60]
						Add 1 mM AgNO_3_ to 25 mL extract heated at 70 °C for 65 min with continuous stirring	Ag	5.54		
*Trapa natans* var. *bispinosa* Roxb.	Peel	Phenolic compounds (gallic acids, quinones)	-	-	Water	Add 0.025 M HAuCl_4_ to extract incubated at 40–60 °C	Au	25 ± 2	Spherical	[87]
						Add 1 mM AgNO_3_ to 25 mL extract incubated at 40–60 °C	Ag	15 ± 2		
*Asparagus* *racemosus*	Root	Phenolics, flavonoids, spiroketal compounds, steroids, reducing sugar, amines, carboxylic acid	-	FTIR	Ethyl acetate	Add 10 mL extract to 50 mL 1 mM HAuCl_4_ irradiated at microwave 700 w and 2.45 GHz for 20 min, incubated 24 h	Au	10–50	Spherical	[65]
						Add 10 mL extract to 50 mL 1 mM AgNO_3_ irradiated at microwave 700 w and 2.45 GHz for 20 min, incubated 24 h	Ag			
*Chrysopogon* *zizanioides*	Leaf	Alkaloids, phytosterols	-	Phytochemical screening, FTIR	Water	Add 10 mL extract to 10 mL 1 mM HAuCl_4_ incubated in 150 rpm rotary shaker in the dark for 2 h	Au	123–138	Cubic	[157]
						Add 10 mL extract to 10 mL 1 mM AgNO_3_ incubated in 150 rpm rotary shaker in the dark for 30 min	Ag	85–110		
*Memecylon* *umbellatum*	Leaf	High saponins, phenolic compounds, protein, quinones	-	Phytochemical screening, FTIR	Water	Add 15 mL extract to 10 mL 1 mM HAuCl_4_ incubated in 150 rpm shaker in the dark for 1 h	Au	15–25	Spherical, triangular, hexagonal	[158]
						Add 15 mL extract to 10 mL 1 mM AgNO_3_ incubated in 150 rpm shaker in the dark for 3 h	Ag	15–20	Spherical	
*Platycodon* *>grandiflorum*	Radix	Triterpenoidal platycodon saponin	-	FTIR	Water	Add 0.05% fraction to 0.2 mM HAuCl_4_ incubated at room temperature for 5 min	Au	14–15	Spherical (major), triangular (minor)	[124]
						Add 0.01% fraction to 0.8 mM AgNO_3_ incubated at 80 °C for 3 h and then at room temperature for 21 h	Ag	17–18	Spherical	
*Coleus forskohlii*	Root	Phenolic compounds	-	FTIR	Water	Add 1 mL extract to 0.4 mL 0.1 mM HAuCl_4_ at pH 7	Au	10–30	Spherical	[66]
						Add 1 mL extract to 0.4 mL 1 mM AgNO_34_ at pH 7	Ag	5–35	Elliptical	
	Root	Forskolin, proteins	-	FTIR	Water	Add 5 mL extract to 10 mL 1 mM boiled HAuCl_4_ stirred at 80 °C for 15 min	Au	15–35	Hexagonal	[67]
						Add 5 mL extract to 10 mL 1 mM AgNO_34_ stirred at 80 °C for 15 min	Ag	35–55	Face-centred cubical	
*Memecylon edule*	Leaf	Saponin	-	FTIR	Water	Add 15 mL extract to 10 mL 1 mM HAuCl_4_ incubated in 150 rpm shaker in the dark for 1 h	Au	10–45	Triangular, circular, hexagonal	[159]
						Add 15 mL extract to 10 mL 1 mM AgNO_3_ incubated in 150 rpm shaker in the dark for 3 h	Ag	50–90	Square	
*Mussaenda* *glabrata*	Leaf	Alkaloids, tannins, flavonoids, steroids	-	FTIR	Water	Mix 1 mM HAuCl_4_·3H_2_O with diluted extract (9:1) incubated at room temperature for 5 min	Au	10.59	Spherical	[68]
						Add 1 mM AgNO_3_ with diluted extract (9:1) incubated at room temperature for 10 min	Ag	51.32		
*Stereospermum chelonoides*	Root bark	Polyphenolic compound (lignans)	-	FTIR	Water	Mix 90 mL 1 mM HAuCl_4_/AgNO_3_ to 10 mL extract irradiated at microwave for 1 min	Au	27.19 ± 5.96	Spherical	[69]
							Ag	49.77 ± 11.64		
*Rivea* *hypocrateriformis*	Aerial part	Polyphenols	-	FTIR	Water	Add 20 mL extract to 50 mL 1 mM HAuCl_4_/ AgNO_3_ irradiated at microwave 700 w and 2.45 GHz for 7 min, incubated for 24 h	Au	20–30	Spherical	[70]
							Ag			
*Gloriosa superba*	Leaf	Glycosides, water soluble tannins	-	FTIR	Water	Add 5 mL extract to 100 mL 1 mM HAuCl_4_ at pH 2.26 for 10 min	Au	20–50	Triangular, spherical	[58]
						Add 5 mL extract to 100 mL 1 mM AgNO_34_ at pH 4.35 for 10 min	Ag			
*Glycyrrhiza* *uralensis*	Root	Flavonoids, polyphenols, glycyrrhizin	-	FTIR	Water	Add 1 mM HAuCl_4_·3H_2_O to extract incubated at 80 °C for 4 min	Au	10–15	Spherical	[71]
						Add 1 mM AgNO_3_ to extract incubated at 80 °C for 40 min	Ag	5–15		
*Aerva lanata*	Leaf	Polyphenols, flavonoids, alkaloids, proteins, sugars, tannins	-	FTIR	Water	Add 10 mL extract to 10 mL 10 mM HAuCl_4_ (final concentration 1 mM) irradiated at microwave 800 w and 2.45 GHz for 90 s	Au	10–30	Spherical, hexagonal, triangular plate	[117]
						Add 10 mL extract to 90 mL 1 mM AgNO_3_ irradiated at microwave 800 w and 2.45 GHz for 1 min	Ag	10–34	Spherical	
*Dendropanax morbifera*	Leaf	Polysaccharides	-	-	Water	Add 1 mM HAuCl_4_·3H_2_O to 1:9 diluted extract incubated at 80 °C for 3 min	Au	10–20	Polygonal, hexagonal	[90]
						Add 1 mM AgNO_3_ to 1:9 diluted extract incubated at 80 °C for 1 h	Ag	100–150	Polygonal, hexagonal, triangle	
*Angelica* *pubescens* Maxim	Root	Flavonoids, phenols, sesquiterpenes	-	FTIR	Water	Add 7 mM HAuCl_4_·3H_2_O to 70% diluted extract incubated at 80 °C for 10 min	Au	10–30	Spherical icosahedral	[140]
						Add 5 mM AgNO_3_ to 50% diluted extract incubated at 80 °C for 50 min	Ag	20–50	Quasi-spherical	
*Amorphophallus paeoniifolius*	Tuber	Phenolic compounds (flavonoid–quercetin)	-	FTIR	Water	Mix 0.8 mM HAuCl_4_·3H_2_O with extract (1:4) incubated at falcon tube for 1 h	Au	13.3	Spherical, polygonal	[57]
						Mix 0.01 mM AgNO_3_ with extract (1:1) incubated at falcon tube and exposed to sunlight for 2–3 min	Ag	22.48		
*Parkia* *roxburghii*	Leaf	Proteins	-	FTIR	Water	Mix 1 g leaf powder with 100 mL 1 mM HAuCl_4_/AgNO_3_ aqueous solution stirred at room temperature for 12 h	Au	5–25	Spherical	[72]
							Ag	5–25	Quasi-spherical	
*Murraya koenigii*	Leaf	Polyphenols, flavonoids, alkaloids		FTIR	Water	Mix 5 mL extract with 25 mL 1 mM HAuCl_4_ aqueous solution stirred vigorously at 300 K for 2 min	Au	20	Spherical, triangle	[160]
						Mix 15 mL extract with 100 mL 1 mM AgNO_3_ boiling aqueous solution, boiling continued for 1 min	Ag	10	Spherical	
*Mentha* *longifolia*	Leaf	Phenolic flavonoids, tannins, saponins and monoterpenes, alkaloids	-	Phytochemical screening, FTIR	Methanol	Mix 1 mM HAuCl_4_/AgNO_3_ with extract (1:1) stirred at 70 °C	Au	10.23 ± 2	Oval	[73]
							Ag	13.45 ± 2		
*Anacardium* *occidentale*	Leaf	Proteins	-	FTIR	Water	Mix 12 mg leaf powder with 30 mL 0.59 mM HAuCl_4_ stirred for 1 min and filtered	Au	17	Spherical	[155]
		Polyols, gallic acid, water soluble tannins	-			Mix 5 mg leaf powder with 30 mL 0.59 mM AgNO_3_ stirred for 1 min and filtered	Ag	15.5		
*Prunus serrulata*	Fruit	Phenolic compounds	-	FTIR	Water	Add HAuCl_4_·3H_2_O to 30 mL 1:9 diluted extract (final concentration 1 mM) incubated at 80 °C for 30 s	Au	20–50	Hexagonal	[112]
		Proteins	-			Add AgNO_3_to 30 mL 1:9 diluted extract (final concentration 1 mM) incubated at 80 °C for 50 min	Ag	20–100	Spherical	
*Zingiber* *officinale*	Root	Ascorbic acid, oxalic acid	-	FTIR	Water	Mix 5 mL extract with 1 mM HAuCl_4_/AgNO_3_ and add 45 mL Ultrapure water at pH alkaline, incubated for 10 h	Au	5–20	Spherical, irregular shape (hexagonal, triangular, truncated triangular)	[74]
							Ag	10–20	Spherical	
*Indigofera* *tinctoria*	Leaf	Phenolic compounds, tannins, alkaloids, saponins, flavonoids, amino acids, carbohydrates, glycosides, steroids	-	FTIR	Water	Add 10 mL extract to 90 mL 1 mM HAuCl_4_ irradiated at microwave 800 w and 2.45 GHz for 30 s	Au	6–29	Spherical, hexagonal, triangular	[75]
						Add 10 mL extract to 90 mL 1 mM AgNO_3_ irradiated at microwave 800 w and 2.45 GHz for 60 s	Ag	9–26	Spherical	
*Bauhinia* *purpurea*	Leaf	Polyphenols	-	FTIR	Water	Add extract to 1 mM HAuCl_4_ (1:10) irradiated at microwave 800 w and 2.45 GHz for 30 s	Au	20–100	Triangular, hexagonal, nanorods	[76]
						Add extract to 1 mM AgNO_3_ (1:10) irradiated at microwave 800 w and 2.45 GHz for 60 s	Ag	20–100	Spherical	
*Cibotium* *barometz*	Root	Flavonoids, phenolic acids, fatty acid	-	FTIR	Water	Add HAuCl_4_·3H_2_O to 5 mL extract diluted with 25 aquadest (final concentration 1 mM), incubated at 80 °C for 50 min	Au	5–20	Spherical	[77]
						Add AgNO_3_ to to 5 mL extract diluted with 25 aquadest (final concentration 1 mM), incubated at 80 °C for 1 h	Ag	5–40		
*Jasminum* *sambac*	Leaf	Polyphenols, flavonoids, terpenoids	-	FTIR	Water	Add 10 mL extract to 50 mL 1 mM HAuCl_4_ irradiated at microwave 700 w and 2.45 GHz for 90 s	Au	20–0	Spherical	[78]
						Add 10 mL extract to 50 mL 1 mM AgNO_3_ irradiated at microwave 700 w and 2.45 GHz for 3 min	Ag			

Abbreviations: HPLC = high-performance liquid chromatography; UV-Vis = ultraviolet-visible spectroscopy; LC-MS = liquid chromatography-mass spectrometry; FTIR = Fourier transform infrared.

**Table 9:** Effect of concentration on the characteristics of AuNPs and AgNPs.

Plant	Type of NP	Optimum Quantity (OQ)	Conditions		Ref.
			Before Reaching OQ	After Reaching OQ	
*Anacardium* *occidentale* leaf	Au	12 mg	↑ Quantity (5–12 mg) causing ↓ λmax = ↓ Particle size	Not measured	[155]
	Ag	5 mg	↑ Quantity (2–5 mg) causing ↓ λmax = ↓ Particle size	↑ Quantity (10 mg) causing ↑ λmax = ↑ Particle size	
*Panax ginseng* fruit	Au, Ag	5%	↑ Quantity (1–5%) causing ↓ λmax = ↓ Particle size	↑ Quantity (6–8%) causing ↑ λmax = ↑ Particle size	[61]
Siberian ginseng (*Eleutherococcus senticosus*) stem	Au, Ag	Proportion metal ions to extract 1:1	Not measured	↑ Quantity (1:2, 1:3) causing ↑ λmax = ↑ Particle size	[39]
Fenugreek (*Trigonella* *foenum-graecum*) seed	Au	3 mL	↑ Quantity (0.5–3 mL) causing ↓ λmax = ↓ Particle size	Not measured	[165]

Note: ↑ = increasing effect, ↓= decreasing effect.

## Data Availability

Data sharing not applicable.
